# Standardized hypoxia-reoxygenation protocol to assess posthypoxic neurobehavioral impairments and molecular mechanisms in *Drosophila melanogaster*

**DOI:** 10.1016/j.xpro.2022.101634

**Published:** 2022-08-18

**Authors:** Jennifer Jung, Aaron T. Fehr, Aaron Voigt, Pardes Habib

**Affiliations:** 1 Department of Neurology, Medical Faculty, RWTH Aachen University, 52074 Aachen, Germany; 2 JARA BRAIN Institute Molecular Neuroscience and Neuroimaging, Forschungszentrum Jülich GmbH and RWTH Aachen University, 52074 Aachen, Germany

**Keywords:** Metabolism, Model organisms, Neuroscience, Behavior

## Abstract

Hypoxia plays a pivotal role in the pathogenesis of major causes of mortality such as cerebral ischemia. Here, we present a standardized protocol for the induction of global hypoxia and reoxygenation in *Drosophila melanogaster*, with details on subsequent analysis of mortality, neurobehavioral impairments, and molecular mechanisms. This protocol emphasizes the importance of controlling and monitoring specific environmental parameters to ensure reproducible results. It also highlights profound differences that can arise from variations in the age and genotype of the flies.

For complete details on the use and execution of this protocol, please refer to Habib et al. (2021).

## Highlights

• Procedures to induce hypoxia in *Drosophila* followed by reoxygenation
• Details on the data analysis to assess posthypoxic neurobehavioral impairments
• Controlling of environmental conditions to ensure reproducibility of hypoxia protocols
• Protocol needs to be modified according to the age and genotype of the flies

## Abstract (editor highlights)

Publisher’s note: Undertaking any experimental protocol requires adherence to local institutional guidelines for laboratory safety and ethics.

## Graphical abstract

**Figure:**
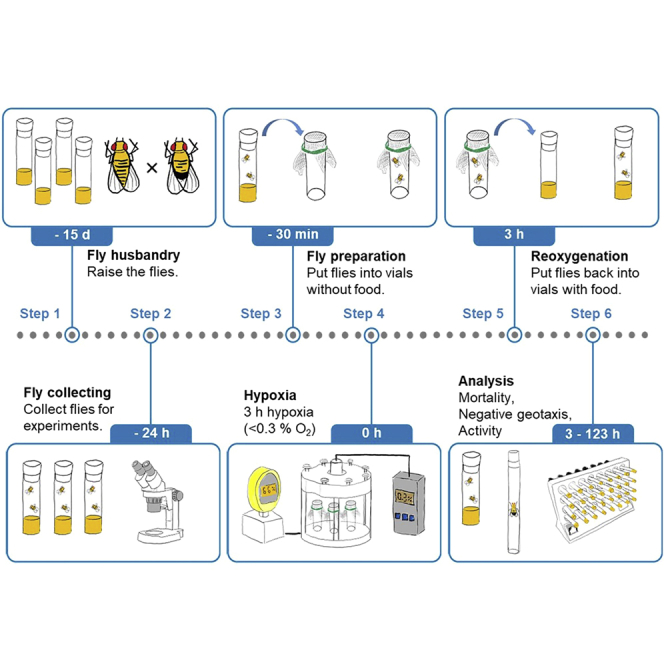

## Before you begin

The protocol below describes the specific steps for 1–5 days old male Canton-S wild-type flies subjected to 2.5 h of severe hypoxia (< 0.3% O_2_) at 29°C, 50%–70% humidity and atmospheric pressure, followed by reoxygenation periods of up to 120 h. A hypoxia duration was chosen in this example as it caused a mortality rate of approximately 50% (Figures 4B–4D), thus resulting in a sufficient impact of hypoxia while providing a reasonable number of viable flies for analysis during the reoxygenation period. Besides that, we have also used this protocol for different hypoxia durations (1–6 h), temperatures (e.g., 18°C, 23°C), pressures (e.g., 300 mbar positive pressure) and humidity levels (e.g., 10%–20% humidity) in various male and female *Drosophila melanogaster* genotypes (e.g., Oregon-R, *Trap1*^*+/−*^, *Trap1*^*−/−*^) of different ages (e.g., 0–1 day old).

### Fly husbandry

**Timing: 13 days before hypoxia**

1. Raise the flies on standard cornmeal food in an incubator at specified conditions as described in the following.
  a. Keep the ratio between female and male flies 3:1 (e.g., 12 female and 4 male flies per vial).
  b. Keep the ambient relative humidity between 50%–70%.
  c. Keep the temperature at 29°C (the F1 generation will hatch after approx. 8 days).
  d. Keep the flies in a 12 h light/dark cycle.
2. After introduction of the parental flies into the vial, allow them to lay eggs for 5 days, before clearing the vials (Figure 1).

**Figure 1:**
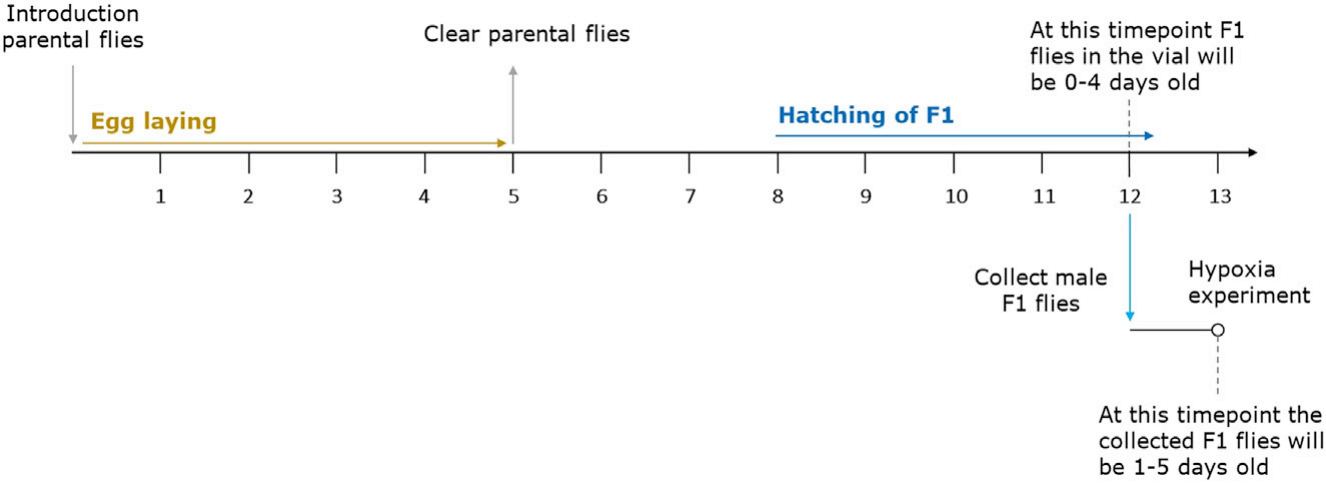
Schematic illustration showing the timeline from introduction of parental flies to the hypoxia experiment The timeline shows all important milestones involved in fly husbandry and collection for flies kept at 29°C.
3. Once the F1 generation has started hatching, let it hatch for 4 more days before collecting them for the hypoxia experiment (Figure 1).

**CRITICAL:** When clearing the vials in step 2, make sure that no parental flies are left in the vial. Clearing of the parental flies will prevent overcrowding and allow age-matching of the F1 generation.

### Fly collecting

**Timing: 24 h before hypoxia; 2 h**

4. Collect the age-matched male F1 flies (0–4 days old) 24 h before the hypoxia experiment (4 days after start of hatching) (Figure 1) into vials with fresh food. For each experimental group/condition we recommend collecting three vials of 20 male flies (in this example we compared hypoxia (0_2_ < 0.3%) and normoxia (0_2_ = 21%) and collected in total 6 vials with 20 male flies each).
  a. Use CO_2_ anesthesia and forceps or a paint brush to collect and transfer the flies.
  b. Place groups of 20 male flies into each new vial.
5. Keep flies as described above until hypoxia experiment.

**Figure 2:**
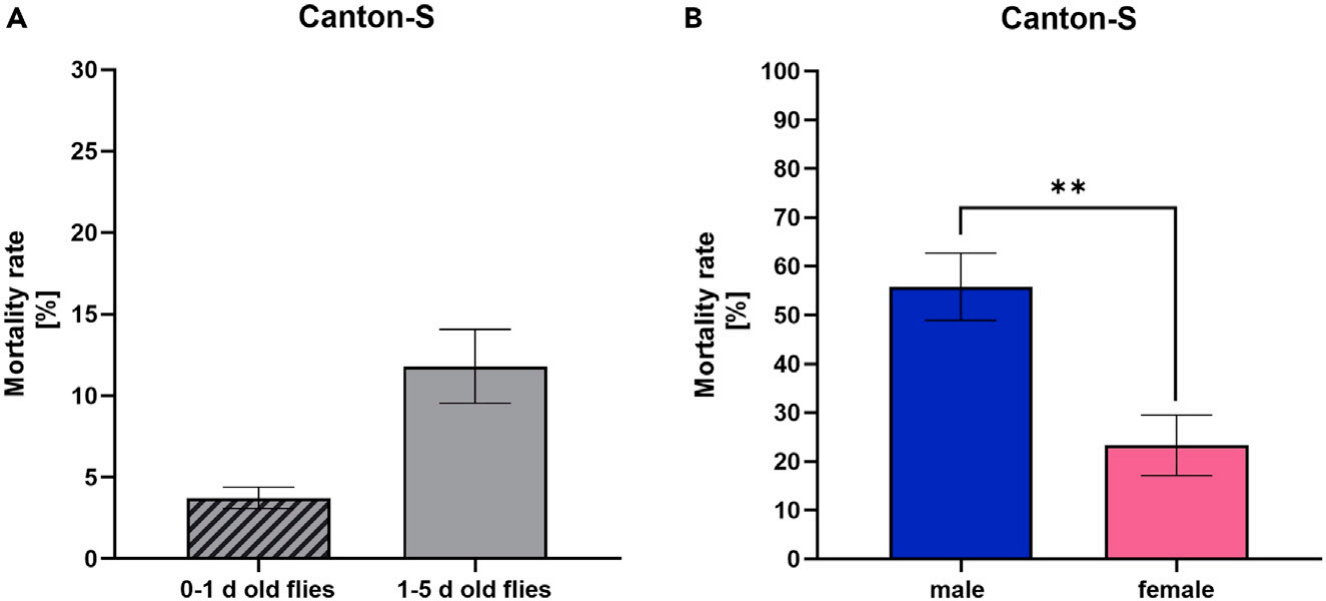
Posthypoxic mortality of Canton-S of different ages and gender (A) Mortality rates of 0–1 day old and 1–5 d old male Canton-S flies after 3 h of severe hypoxia (< 0.3% O_2_) at 23°C, 50%–70% humidity and atm. pressure, followed by 120 h of post-hypoxic reoxygenation. (B) Mortality rates of 1–5 d old male or female Canton-S flies after 2.5 h of severe hypoxia (< 0.3% O_2_) at 29°C, 50%–70% humidity and atm. pressure, followed by 120 h of post-hypoxic reoxygenation. Data are shown as means ± SEM of 3 independent experiments, including 3 technical replicates per experiment (total of 180 flies per condition. Unpaired t-test. p = 0.0031.

**CRITICAL:** The selection of flies is based on the purpose of the experiment. It is necessary to use age- and gender-matched flies. Flies of different age or gender can respond differently to the hypoxic stimulus (Figures 2A and 2B). Note that the genotype of flies may affect hypoxia tolerances. It cannot be assumed that a specific hypoxic stimulus will have the same impact on the outcome of one fly strain as on another.

## Key resources table

**Table:** 

REAGENT or RESOURCE	SOURCE	IDENTIFIER
**Chemicals, peptides, and recombinant proteins**		

Agar-Agar	Gewürzmühle Brecht	00162-0500
Brewer’s yeast	Gewürzmühle Brecht	03462-2500
Soy flour	Gewürzmühle Brecht	N/A
Sugar beet syrup	Grafschafter	1905
Nipagin (Tegosept)	Apex	Cat# 20-259
Propionic acid	Carl Roth GmbH	6026.3
Yeast Peptone - Agar	Carl Roth GmbH	X965.3
Sucrose (D(+)-Saccharose)	Carl Roth GmbH	4621.1

**Experimental models: Organisms/strains**		

*Drosophila melanogaster* wild-type strain Canton-S, male flies, age: 1–5 d	Bloomington Drosophila Stock Center	#64349; RRID:BDSC_64349

**Software and algorithms**		

DAMSystem3 Software	TriKinetics	https://www.trikinetics.com/
Microsoft Excel	Microsoft	https://microsoft.com/microsoft-365/excel
Graphpad Prism 8	GraphPad	https://graphpad.com/scientific-software/prism/

**Other**		

Hypoxia chamber	self-constructed	N/A
Plastic vials	Dutscher	789009
Gauze swabs 5 × 5 cm	Fuhrmann	31111
Rubber Band	N/A	N/A
Greisinger GOX 100 T O2-Sensor	Greisinger	H59.0.22.6C-06
Fluke 700RG06 100 PSIG pressure gauge	Fluke	700RG06
Habor Thermo-Hygrometer sensor for temperature and humidity	Habor	N/A
DAM2 *Drosophila* Activity Monitor	TriKinetics	Cat#DAM2
Cables for DAM2 System	TriKinetics	Cat#CAB2
Glass tubes	TriKinetics	Cat#PPT5x65

## Materials and equipment

**Table:** Fly food for husbandry and hypoxia / normoxia

Reagent	Final concentration	Amount
Agar-Agar	0.675%	135 g
Brewer’s yeast	1.8%	360 g
Soy flour	8%	1,600 g
Sugar beet syrup	2.2%	440 g
Nipagin (30%)	0.1875%	125 mL
Propionic acid	0.63%	126 mL
H_2_O	N/A	20 L
**Total**	**N/A**	**20 L**

All chemicals are stored at room temperature. Fly food can be stored at 18°C for a maximum of 1 week.

**Table:** Fly food for DAM

Reagent	Final concentration	Amount
Yeast Peptone - Agar	2%	1 mg
Sucrose	4%	2 mg
ddH_2_O	N/A	50 mL
**Total**	**N/A**	**50 mL**

Boil in microwave to dissolve agar. Fill liquid food into the DAM vials (∼1.5 cm on one end).

Let the food dry overnight at 18°C.

Fly food for DAM should be prepared no earlier than 1 day before the experiment.

## Step-by-step method details

### Fly preparation for hypoxia

**Timing: 30 min**

In this step the flies are prepared for the hypoxia and transferred into the designated hypoxia vials.

1. Transfer the collected F1 male flies (now 1–5 d old) into vials for hypoxia.
  a. Anesthetize the flies in each vial using CO_2_.
  b. Transfer all 20 flies of each vial into an empty vial without food.
  c. Use gauze to cover the open side of the vial to guarantee an effective gas exchange.
  d. Fix the gauze with a rubber band.
2. Prepare the flies for the normoxia control condition.
  a. Keep the flies in the same vials with food.
  b. Anesthetize the flies in each vial using CO_2_.
  c. Exchange the plug and cover the open side of the vial with gauze and rubber bands.

**CRITICAL:** Keep the CO_2_ anesthesia time as short as possible as pre-hypoxic anesthesia might influence the adaptation of the flies to the hypoxia. Use the same anesthesia times for hypoxia and normoxia conditions. Before hypoxia induction make sure all flies have recovered from the CO_2_ anesthesia in their vial. We recommend a recovery period of at least 3 min.

***Note:*** The normoxia flies should be kept in vials with food to prevent stress from starvation. However, flies subjected to hypoxia should be kept in vials without food to prevent them from sticking to the food when unconscious during hypoxia, as this could negatively bias mortality rates. Since the flies lose consciousness shortly after exposure to hypoxia, they do not require food during hypoxia.

### Hypoxia induction

**Timing: 2.5 h**

***Note:*** Timing depends on hypoxia duration, which is adjustable depending on the experiment and the genotype used.

In this step the flies are subjected to a hypoxic stimulus for a designated period of time under controlled and monitored environmental conditions.

**Figure 3:**
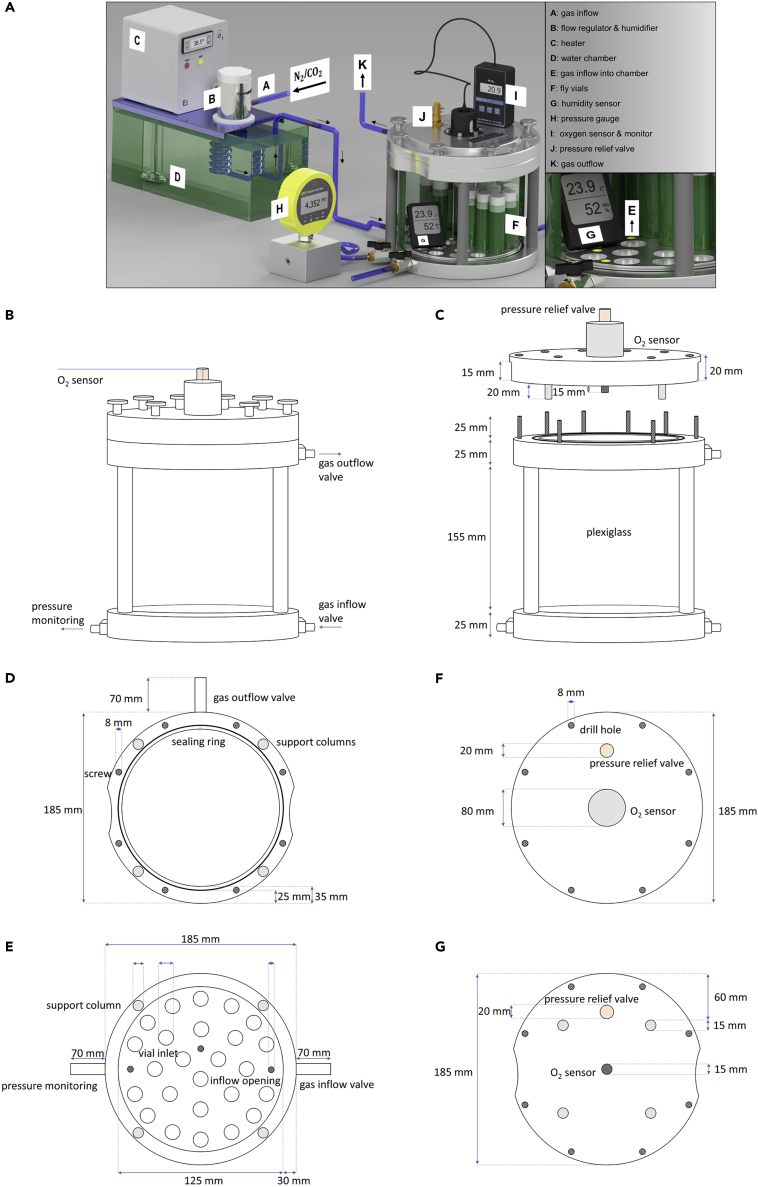
Technical illustration and details of the hypoxia chamber (A) Complete setup of the hypoxia chamber with air heater and humidifier, pressure sensor, oxygen sensor and temperature and humidity sensor. This graph was taken from (Habib et al., 2021). (B) Technical illustration of the self-designed hypoxia chamber. (C) Technical illustration and dimensions of the chamber and the lid. (D) Technical illustration and dimensions of the top of the chamber. (E) Technical illustration and dimensions of the bottom of the chamber. (F and G) Technical illustration and dimensions of the lid. View from above (F) and view from below (G).
4. Place the temperature and humidity sensor inside the chamber alongside the vials.
5. Close and secure the lid tightly. Troubleshooting 1.
6. Attach the pressure gauge to the chamber.
7. Attach the oxygen sensor to the top of the chamber.
8. Close any unused valves of the chamber and open the gas outflow valve.
9. Open the gas entry valve and start the nitrogen gas flow to flood the hypoxia chamber.
  a. Pass the gas through a heated water container to humidify the gas.
  b. Keep the inflow at a controlled and steady rate. We recommend an air flow rate of ∼ 15 L/min.
10. Flood the hypoxia chamber with the humidified nitrogen until an oxygen concentration of < 0.3% is reached, then stop the gas flow, close the gas entry valve and then the gas outflow valve. During hypoxia induction, monitor humidity and pressure in the chamber. Troubleshooting 2.
11. Place the hypoxia chamber in an incubator (29°C, 50%–70% humidity, atm. pressure) for the experiment. Monitor oxygen levels, temperature, humidity and pressure regularly during the hypoxia.
12. Place the vials containing the normoxia control group next to the hypoxia chamber in the incubator for the duration of the hypoxia.

***Note:*** Other gasses besides nitrogen, such as CO_2_, can also be used to induce hypoxia.

3. Place the vials into the hypoxia chamber. A detailed description of such a hypoxia chamber can be found in Figure 3.

**Figure 4:**
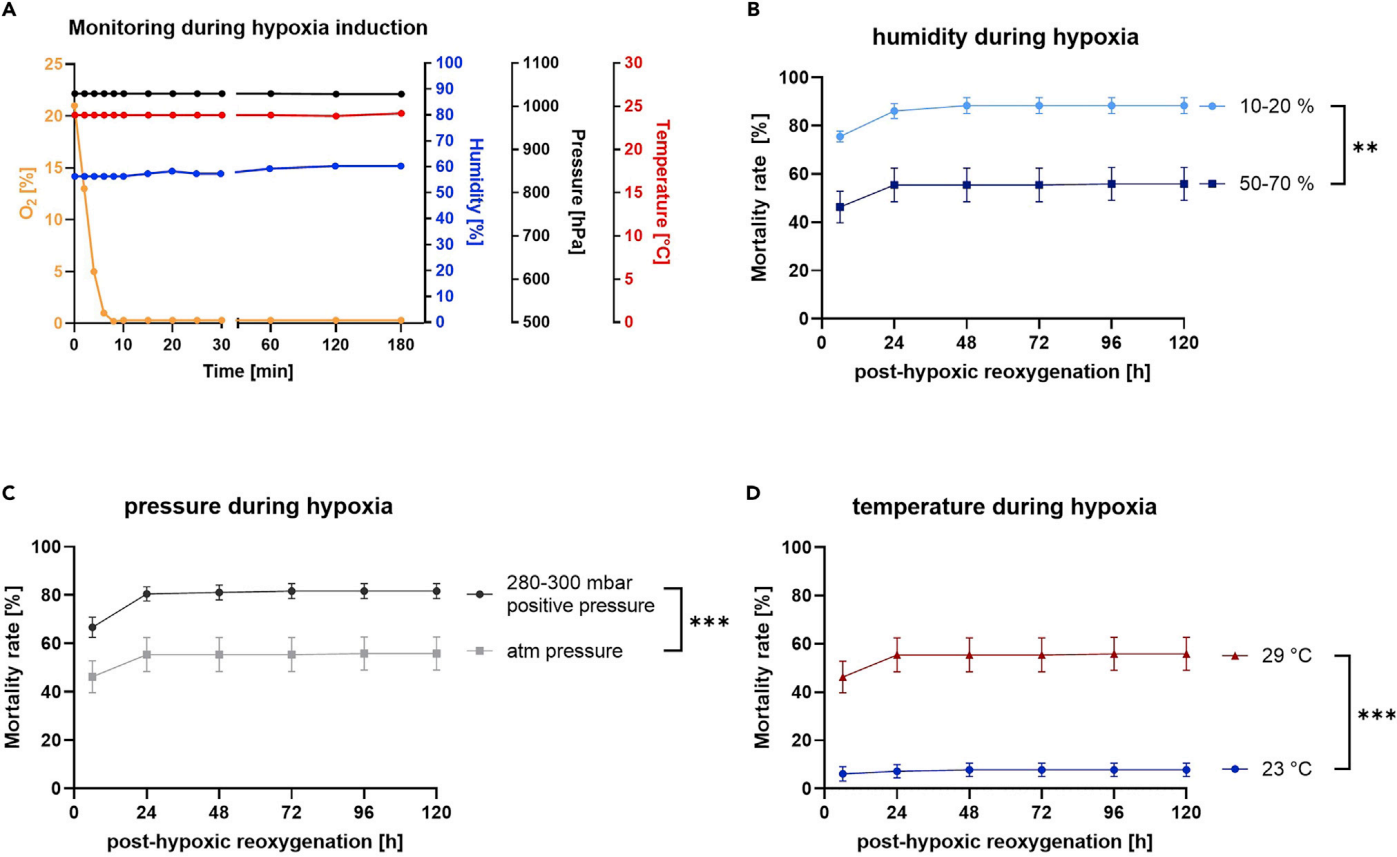
Posthypoxic mortality of 1–5 d old male Canton-S after 2.5 h of severe hypoxia (< 0.3% O_2_) under different environmental conditions during a reoxygenation period of 120 h (A) Graph displaying the O_2_ levels, humidity, pressure and temperature in the hypoxia chamber during 3 h of hypoxia. (B) Posthypoxic death rates of Canton-S wildtype flies during a reoxygenation period of 120 h after 2.5 h of hypoxia (29°C, atm. pressure) with either 10%–20% or 50%–70% of humidity in the hypoxia chamber. (C) Posthypoxic death rates of Canton-S wildtype flies during a reoxygenation period of 120 h after 2.5 h of hypoxia (29°C, 50%–70% humidity) with either atmospheric pressure or 280–300 mbar positive pressure. (D) Posthypoxic death rates of Canton-S wildtype flies during a reoxygenation period of 120 h after 2.5 h of hypoxia (50%–70% humidity, atm. pressure) at either 23°C or 29°C. Data are shown as means ± SEM of 3 independent experiments, including 3 technical replicates per experiment (total of 180 male flies for each condition). Multiple t-tests. ∗∗p<0.01; ∗∗∗p<0.001. The graphs B, C and D were modified from (Habib et al., 2021).

**CRITICAL:** Make sure that the gas outflow valve is at the top of the chamber as nitrogen displaces oxygen upwards. When using different gases, take their density into consideration and adjust flooding from the top or the bottom accordingly. Monitoring and maintaining the environmental factors temperature, humidity and pressure at their set value is crucial to ensure reproducible results, as these parameters have an impact on the outcome of the flies after hypoxia (Figures 4A–4C). Therefore, it is important to humidify the gas before flooding, to set a specific temperature and to make sure the hypoxia chamber is equipped with a pressure relief valve to prevent overpressure. It is vital for the reproducibility of the results to keep these environmental parameters the same throughout all hypoxia experiments.

### Reoxygenation

**Timing: 5 min + reoxygenation period**

***Note:*** Timing depends on the duration of the reoxygenation period. The duration of the reoxygenation period can depend on the chosen readout (see 17. Readout recommendations).

In this step, hypoxia is terminated and the flies are transferred to a normoxic environment for a designated period of time under controlled and monitored conditions.

13. Open the hypoxia chamber and take out the fly vials.
14. Remove the rubber bands and gauze and transfer the flies from each vial into new vials with fresh food.
15. Keep flies in the incubator at 29°C, 50%–70% humidity and atm. pressure as described in section “fly husbandry” for the entire reoxygenation period.
16. Repeat steps 14 and 15 with the normoxia control flies.

***Note:*** Place vials on the side until flies regain movement ability to prevent the flies from sticking to the food.

17. Readout recommendations:
  a. Mortality rate assessment: To assess the mortality rate during a reoxygenation period, count the number of dead flies per vial 6, 24, 48, 72, 96 and 120 h after hypoxia.
    ***Note:*** The presence of dead flies may trigger a stress response in the remaining flies. However, removal of the dead flies entails recurrent exposures to CO_2_-anesthesia, which could influence outcome after hypoxia. This should be taken into consideration for further analyses.
  b. Negative geotaxis assay: Assess the post-hypoxic evoked climbing ability at 3, 6, 24, 48, 72, 96 and 120 h of reoxygenation under normal lighting conditions.
    i. Transfer the groups of flies into empty plastic vials with a mark at 8 cm.
    ii. Tap the flies to the bottom.
    iii. Assess the percentage of vivid flies that are able to reach the 8 cm mark in 10 s.
    iv. Repeat step ii. and iii. 10 times with 50 s recovery time between each repetition (Ali et al., 2011).
  c. *Drosophila* activity monitoring: Monitor the post-hypoxic activity of the flies under controlled environmental conditions in a *Drosophila* Activity Monitoring System.
    i. Transfer each fly individually into one food-containing polycarbonate vial (∼ 1.5 cm food height) after the hypoxia using a forceps or a paint brush.
      ***Note:*** As the flies subjected to hypoxia do not regain consciousness until approximately 30 min after hypoxia, no CO_2_ anesthesia is needed for this step. However, normoxia flies need to be anesthetized for the transfer into the DAM-tubes.
    ii. Start the recording in the DAMSystem3 Software. Set the recording frequency to 1 h. The activity is automatically recorded using a light beam passing through the center of each vial. Any interruption of the light beam, e.g., caused by a fly crossing through it, is detected.
      **CRITICAL:** Since the time of day affects fly activity, it is important to keep hypoxia times as well as the start point for the DAM measurements similar for all runs.
    iii. Keep the flies in the DAM system in an incubator set to 29°C, 50%–70% humidity and atm. pressure for a total of 5 days.
    iv. After 5 days, examine every fly individually to determine the total death rate.
      ***Note:*** Lighting conditions can be adjusted depending on the readout you are aiming for. It is possible to assess activity by either exposing the DAM system to constant light over the entire observation period or simulate a day-night rhythm by alternating 12 hours of light and 12 hours of darkness. It is also possible to analyze circadian rhythms through first synchronizing the circadian rhythm for 3 days by exposing the flies to a light-dark rhythm and then assessing the activity patterns during 5 days of constant darkness (Cichewicz and Hirsh, 2018; Schmid et al., 2011).

## Expected outcomes

**Figure 5:**
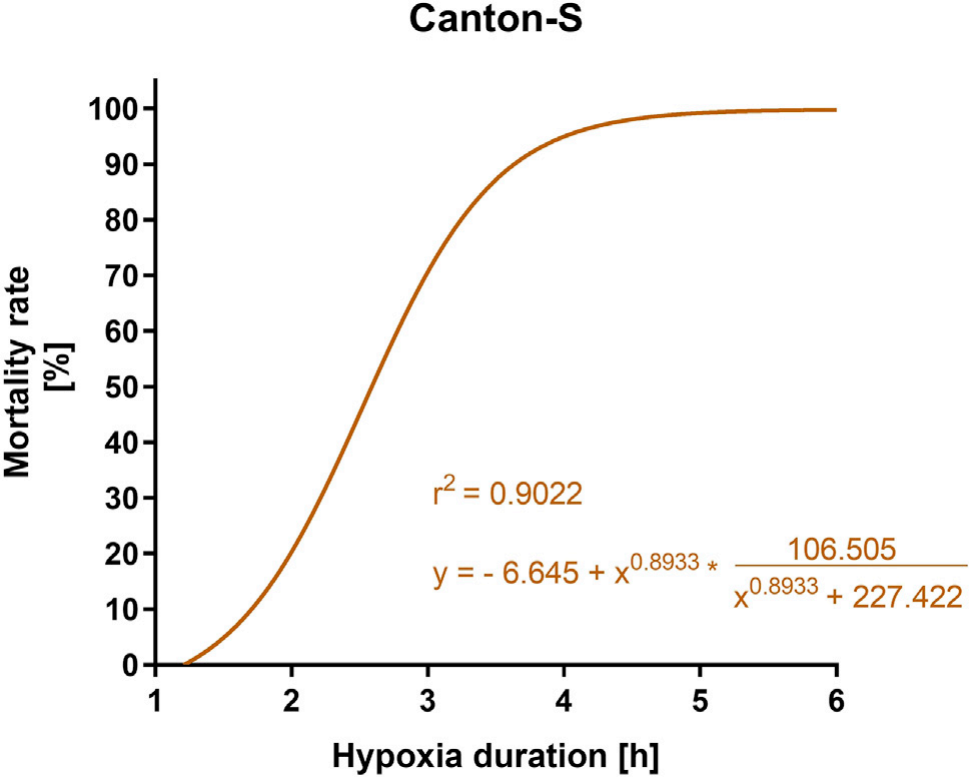
Regression Curve showing the hypoxia duration dependent mortality rate of male 1–5 d old Canton-S flies after a posthypoxic reoxygenation period of 120 h Hypoxia was performed for 1–6 h at < 0.3% O_2_, 29°C, 50%–70% humidity and atm. pressure and the mortality rates were assessed after a reoxygenation period of 120 h. Data of 4 independent experiments, including 3 technical replicates per experiment (total of 240 male flies for each hypoxia duration). This figure was modified from (Habib et al., 2021).

**Figure 6:**
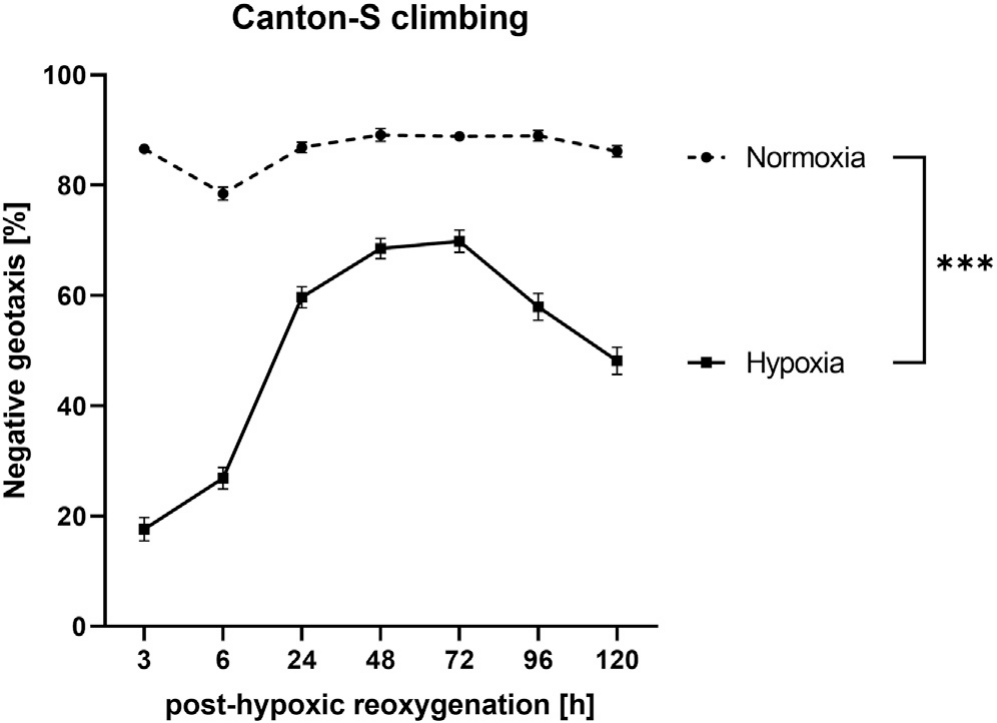
Posthypoxic negative geotaxis of Canton-S flies Negative geotaxis (climbing ability) was assessed during a reoxygenation period of 120 h after 1–5 d old male Canton-S flies were subjected to 2.5 h of severe hypoxia (< 0.3% O_2_) at 29°C, 50%–70% humidity and atm. pressure. Data are shown as means ± SEM of 3 independent experiments, including 3 technical replicates per experiment (total of 180 male flies). Multiple t-test. ∗∗∗p<0.001.

**Figure 7:**
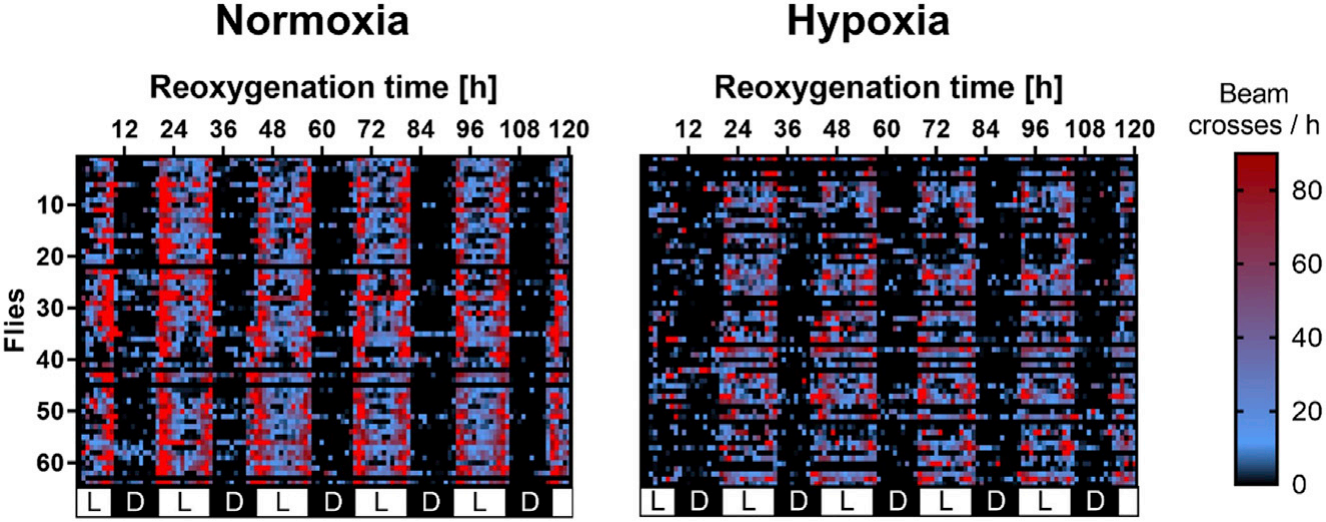
Activity of Canton-S after 2.5 h of severe hypoxia Heatmap displaying the activity of male 1–5 d old Canton-S during a reoxygenation period of 120 h after 2.5 h of severe hypoxia (< 0.3% O_2_) at 29°C, 50%–70% humidity and atm. pressure. The spontaneous activity was assessed in a *Drosophila* Activity Monitor System. Each cell shows the absolute value of beam crosses per hour, which is represented by different colors (black: little to no activity, blue: moderate activity, red: high activity), as indicated in the legend.

Flies subjected to hypoxia are expected to exhibit significantly higher mortality rates compared to normoxic control conditions. The mortality rates are expected to increase with longer hypoxia durations. Moreover, hypoxia leads to posthypoxic neurobehavioral impairments, leading to decreased climbing ability and lower spontaneous activity. As an example, the mortality rate of Canton-S flies after different durations of hypoxia was significantly increased (Figure 5). The climbing ability and the overall spontaneous activity of the Canton-S flies were significantly reduced after hypoxia compared to the normoxic control (Figures 6 and 7). Outcomes can differ depending on the genotype and the experimental setup.

## Quantification and statistical analysis

**Timing: 2–5 h**

For quantification and statistical analysis, Microsoft® Excel® and GraphPad Prism (version 8.4.3, San Diego, CA, USA) can be used.

Below you will find detailed instructions describing how to analyze the obtained data.

### Mortality rate analysis

1. Insert your raw data into a new Microsoft Excel sheet containing the total of flies and the number of dead flies in each vial.
2. Calculate the mortality rate according to the following equation:

$$Mortality\;rate=\frac{\#\;of\;dead\;flies}{Total\;\#\;of\;flies\;in\;the\;vial}*100\;\%$$

3. Repeat steps 1 and 2 for each hypoxia duration individually.
4. Calculate the means of the 3 technical replicates for each condition.
5. Open the GraphPad Prism software and create a new XY table with 4 replicate values.
6. Enter the data into the XY table.
7. Go to “Analyze” and “Nonlinear regression (curve fit)” in the “XY analysis” menu and click “OK”.
8. Select “Logistic growth” under the “Growth curves” menu and click “OK”.
9. Look up the r^2^ value and the formula in the “Results” sheet.

### Negative geotaxis analysis

10. Insert the raw data of the mortality assessment and of the negative geotaxis assessment into a new Microsoft Excel sheet.
11. Calculate the mortality adjusted negative geotaxis for each measured time point individually according to the following equation:

$$Negative\;geotaxis=\frac{\#\;of\;flies\;with\;intact\;negative\;geotaxis}{\;\#\;of\;living\;flies\;in\;the\;vial}*100\;\%$$

### Mean activity analysis

12. Download the DAMFileScan Software on the TriKinetics webpage.
13. Choose your monitor files (∗.txt) and select the time period of data assessment. The bin length should be set to 60 min. Make sure that all data start at the same time point.
14. Save the files.
15. Insert the data into Microsoft Excel.
16. Exclude dead flies.
  a. Create a macro with the following code (in this example we define the point of death as the timepoint, from which the maximum activity is <70 and the mean activity <10 for the rest of the observation period. Moreover, we only define flies as dead, when they die before the last 24 h of observation, as we cannot guarantee that inactivity of <24 h can be interpreted as death.
    Sub Exclude_Dead()
     For c = 11 To 42
     'c for the number of flies represented in the 32 columns after column 10
      For r = 1 To 120
      'r for the number of rows in 120 h of reoxygenation period
       Dim maximum As Integer
       maximum = WorksheetFunction.Max(Range(Cells(r, c), Cells(120, c)))
       Dim mean As Integer
       mean = Application.Average(Range(Cells(r, c), Cells(120, c)))
       If maximum < 70 And mean < 10 And r < 96 Then
       'This line defines the exclusion criteria for dead flies.
        Range(Cells(r, c), Cells(120, c)).Interior.ColorIndex = 3'marks all cells in red after the fly’s death
       End If
      Next
     Next
    End Sub
  b. Run the macro.
  c. Delete the cells that were marked in red by the macro.
17. Calculate the mean of each row. Microsoft Excel automatically ignores the now empty cells, so that the result is not influenced by dead flies.

***Optional:*** It is also possible to plot the exact point of death of the flies.

### Sleep cycle analysis

Sleep cycles can be analyzed by multiple sleep analysis tools, e.g., SCAMP (Donelson et al., 2012) or ShinyR-DAM (Cichewicz and Hirsh, 2018).

## Limitations

The protocol described above requires precise monitoring of the environment of the flies during hypoxia and reoxygenation. A hypoxia chamber capable of keeping constant pressure, humidity and temperature and oxygen levels is needed.

Furthermore, although *Drosophila* is a suitable model to study the impact of hypoxia on both behavioral and molecular outcomes, the limitations regarding the studied pathophysiology should always be kept in mind. For example, while *Drosophila* shares many conserved molecular mechanisms with humans, differences in physiology and the respiratory system should be taken into account. Moreover, one can only expose the fly to a global hypoxia with the protocol at hand, whereas it is not possible to generate a selective hypoxia (e.g., only in the brain). However, it is possible to isolate proteins or mRNA only from the fly heads or body and thus to study the impact on specific parts of the fly.

Lastly, the studied *Drosophila* genotype has a great impact on the readouts, so that we recommend establishing suitable hypoxia durations for every genotype individually.

## Troubleshooting

### Problem 1

Oxygen concentration rises during hypoxia (section “hypoxia induction”, steps 5–11).

### Potential solution

Make sure to check the hypoxia chamber for any leaks before use. It is recommended to verify the integrity of the chamber regularly, by e.g., flooding the hypoxia chamber to set it to a specific oxygen level and then leaving it for one or two days. The oxygen concentration in the chamber should remain constant.

### Problem 2

Oxygen sensor shows varying starting values (section “hypoxia induction”, steps 7–10).

### Potential solution

Make sure to calibrate the oxygen sensor regularly to ensure its reliable function.

## Resource availability

### Lead contact

Further information and requests for resources and reagents should be directed to and will be fulfilled by the lead contact, Pardes Habib (phabib@ukaachen.de).

### Materials availability

This study did not generate new unique reagents. All materials are commercially available. The hypoxia chamber was self-designed, technical schemes can be found in Figure 3.

## Data Availability

All code generated in this study was inserted in the protocol at the relevant step. The authors confirm that all data underlying the findings are fully available without restrictions.
